# Proteomic Analysis of PKCγ-Related Proteins in the Spinal Cord of Morphine-Tolerant Rats

**DOI:** 10.1371/journal.pone.0042068

**Published:** 2012-07-31

**Authors:** Zongbin Song, Qulian Guo, Jie Zhang, Maoyu Li, Chang Liu, Wangyuan Zou

**Affiliations:** 1 Department of Anesthesiology, Xiangya Hospital, Central South University, Changsha, China; 2 Department of Anesthesiology, the Maternal and Child Health Hospital of Hunan Province, Changsha, China; 3 Key Laboratory of Cancer Proteomics of Chinese Ministry of Health, Xiangya Hospital, Central South University, Changsha, China; The James Cook University Hospital, United Kingdom

## Abstract

**Background:**

Morphine tolerance is a common drawback of chronic morphine exposure, hindering use of this drug. Studies have shown that PKCã may play a key role in the development of morphine tolerance, although the mechanisms are not fully known.

**Methodology/Principal Findings:**

In a rat model of morphine tolerance, PKCã knockdown in the spinal cord was successfully carried out using RNA interference (RNAi) with lentiviral vector-mediated short hairpin RNA of PKCã (LV-shPKCã). Spinal cords (L4-L5) were obtained surgically from morphine-tolerant (MT) rats with and without PKCã knockdown, for comparative proteomic analysis. Total proteins from the spinal cords (L4-L5) were extracted and separated using two-dimensional gel electrophoresis (2DGE); 2D gel images were analyzed with PDQuest software. Seven differential gel-spots were observed with increased spot volume, and 18 spots observed with decreased spot volume. Among these, 13 differentially expressed proteins (DEPs) were identified with matrix-assisted laser desorption/ionization-time of flight mass spectrometry (MALDI-TOF MS), comparing between MT rats with and without PKCã knockdown. The DEPs identified have roles in the cytoskeleton, as neurotrophic factors, in oxidative stress, in ion metabolism, in cell signaling, and as chaperones. Three DEPs (GFAP, FSCN and GDNF) were validated with Western blot analysis, confirming the DEP data. Furthermore, using immunohistochemical analysis, we reveal for the first time that FSCN is involved in the development of morphine tolerance.

**Conclusions/Significance:**

These data cast light on the proteins associated with the PKCã activity during morphine tolerance, and hence may contribute to clarification of the mechanisms by which PKCã influences MT.

## Introduction

Morphine is the cornerstone of the management of both cancer pain and postoperative pain. Despite its widespread use, treatment with morphine is often accompanied by the development of tolerance and dependence [1]. Morphine tolerance is characterized by a reduced responsiveness to the drug, which usually manifests as a need to use increasing doses to achieve the desired action; this in turn enhances the severity of non-analgesic side effects. It is well established that morphine exerts its anti-nociceptive effect mainly through activation of the mu opioid receptor (MOR), but that morphine tolerance and physical dependence do not require modification of the MOR [2], [3]. Studies have shown that morphine tolerance is related to adaptation of numerous process in the central nervous system, such as N-methyl-d-aspartate receptors, and neuropeptide and opioid systems, but the mechanisms underlying this phenomenon are still not clearly understood [4], [5], [6]. Recent studies have shed light on the neurobiology of morphine tolerance, and a growing body of evidence suggests that protein kinase C (PKC) plays a key role in the development of morphine tolerance [7], [8], [9].

There is accumulating evidence that PKC activity may be associated with the generation and development of pain, including neuropathic pain and hyperalgesia [10], [11]. Moreover, PKC has been implicated in the development of opioid dependence and tolerance [12]. The PKC family consists of at least 12 isoforms that differ with regard to their structure, substrate requirements, expression and localization. Studies have suggested that the various PKC isoforms play differing physiological roles in morphine tolerance and the other side-effects of this drug [13], [14], [15].The PKC gamma isoform (PKCã) is an auto-inhibitory enzyme that, in the presence of calcium ions, activates second messenger cascades via 1,2-diacyl-glycerol and membrane phospholipids. A growing number of reports have indicated that PKCã is widely distributed throughout the central nervous system (CNS) and plays a major role in the development of morphine tolerance [12], [15], [16], [17]. Li and colleagues reported that chronic intrathecal morphine administration (10 µg twice-daily, for 6 d) in rats induced tolerance to the anti-nociceptive effect of morphine, as well as a time-dependent up-regulation of PKCã within the dorsal horn of the spinal cord [18]. Furthermore, it has been well documented that morphine tolerance in the spinal cord is dependent upon an increase in the local phosphorylating activity of PKC, and that blocking PKC action prevents the expression of morphine tolerance [19]. In a previous study, we found that anti-nociceptive tolerance to chronic morphine administration could be reversed by reducing the expression of PKCã in the spinal cord [20]. However, the molecular and cellular mechanisms underlying these processes are still not fully understood. Moreover, study of PKCã-dependent pathways is often limited by the absence of effective PKCã inhibitors with selectivity for individual isoforms.

RNA interference (RNAi) has been established as a powerful technique with which to investigate gene function. RNAi is a post-transcriptional gene silencing mechanism, where a short double-stranded RNA guides the recognition and cleavage of messenger RNA [21]. Plasmid-based short hairpin RNA (shRNA) is an advanced modification of RNAi, which produces robust and prolonged RNA inhibition and knockdown of the target protein [22], [23]. Lentiviral vectors provide a means to express shRNA and induce stable and long-term gene silencing in both dividing and non-dividing cells [24]. In previous studies, we have developed a highly efficient method for lentiviral-mediated delivery of shRNAs that target PKCγ, allowing *in vivo* gene silencing in the spinal cord of rats and identification of novel targets of PKCã that may be involved in neuropathic pain [20], [25]. Proteomics is an effective platform to globally detect and characterize proteins, and a comparative proteomic approach is a highly efficient method for revealing differences in protein expression between control and treated samples. Most studies examining proteomic changes after morphine administration have focused on the development of morphine dependence, and have concentrated on identifying changes in various brain regions [26], [27], [28]. Only Shui and coworkers have carried out a comparative proteomic study of proteins in the spinal cord of morphine-tolerant (MT) rats [29].

In the present study, we have used two-dimensional gel electrophoresis (2DGE)-based comparative proteomics in combination with an RNAi-mediated gene-silencing technique, to identify novel protein targets involved in the PKCã signaling pathway that might play a role in morphine tolerance The spinal cords (L4-L5) from MT rats, with and without PKCã-knockdown, were used for proteomic analysis. The extracted proteins were separated by 2DGE, and gel image analysis was used to define each differentially expressed proteins (DEP). The protein that was contained in each gel-spot was characterized with matrix-assisted laser desorption/ionization-time of flight mass spectrometry (MALDI-TOF) peptide mass fingerprint (PMF) analysis, and Western blot and immunohistochemical techniques were employed to confirm the expressional changes in the identified proteins.

## Results

### Establishing a MT Rat Model with PKCã-knockdown

**Figure 1:**
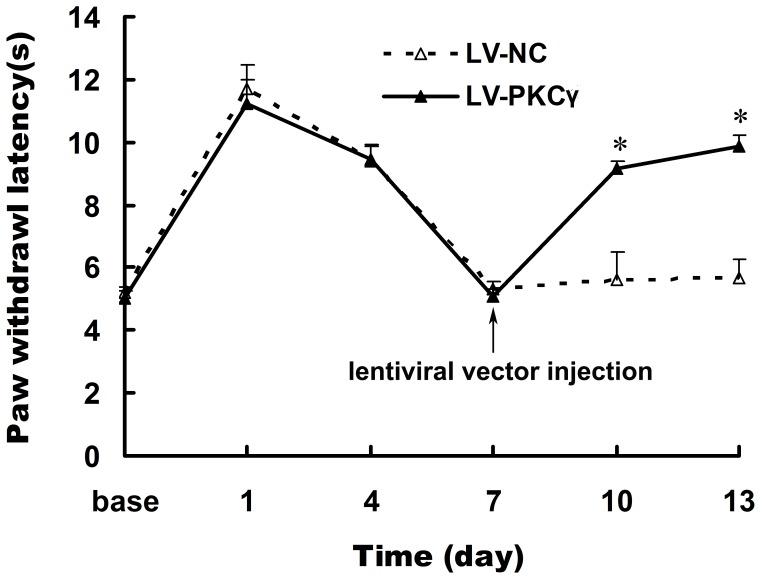
Effects of morphine injections on thermal paw withdrawal latencies (PWLs). The anti-nociceptive effect was measured 30 min after morphine injection. The baseline PWLs were determined before chronic morphine exposure (10 µg i.t., twice daily for 6 days). On day 7, rats were injected intrathecally with LV-NC or LV-shPKCã. Data are presented as mean ± SD. *P<0.05, compared with the corresponding value in the saline control group.

To ensure that all morphine-treated rats used in the experiments displayed morphine tolerance, the latencies of the hind-limb withdrawal response to radiant heat were measured, before and 30 min after injection of 10 µg morphine, every three days. Chronic morphine exposure in rats led to a stable anti-nociceptive tolerance, whereas LV-shPKCã was found to significantly reverse morphine tolerance (Figure 1). During the development of morphine tolerance, there were no differences in the paw withdrawal latencies (PWLs) between LV-NC and LV-shPKCã rats. A single injection of LV-shPKCã significantly reversed morphine anti-nociceptive tolerance on days 10 and 13 of the experimental period, compared with the LV-NC group.

**Figure 2:**
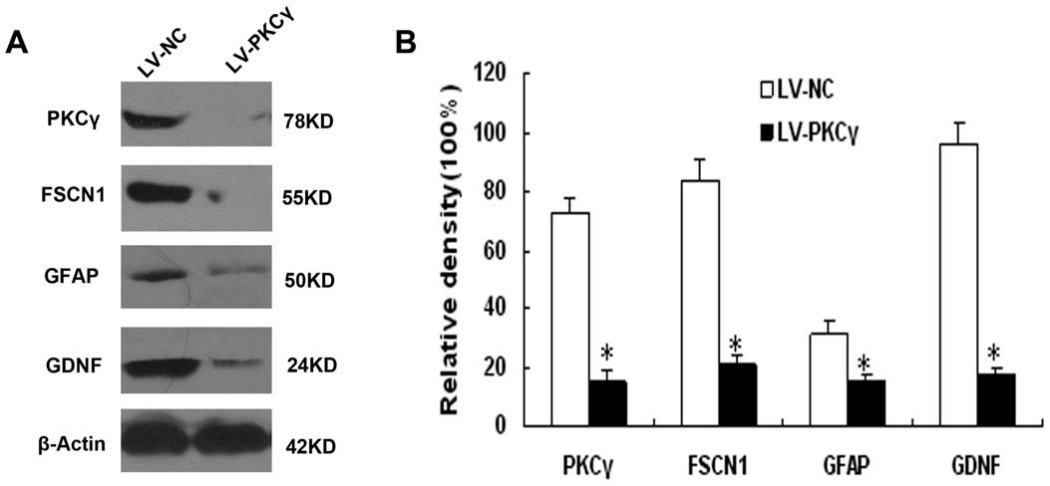
Effect of LV-PKCã treatment on expression of proteins in the spinal cords of morphine-tolerant rats. (A) Western blot detection of PKCã and other differentially expressed proteins. (B) The expression level for each protein was estimated by densitometry, and is shown as a ratio to the loading control, â-actin. Data are presented as mean ± SD. *P<0.05, compared with the corresponding value in the LV-NC group.

Maximal reversal of anti-nociceptive tolerance to morphine was observed 7 days after siRNA injection; therefore, we examined the effect of LV-shPKCã injection on protein expression at day 14. As shown in Figure 2, injection of siRNA significantly down-regulated the expression of PKCã.

### Identification of Differentially Expressed Proteins

**Figure 3:**
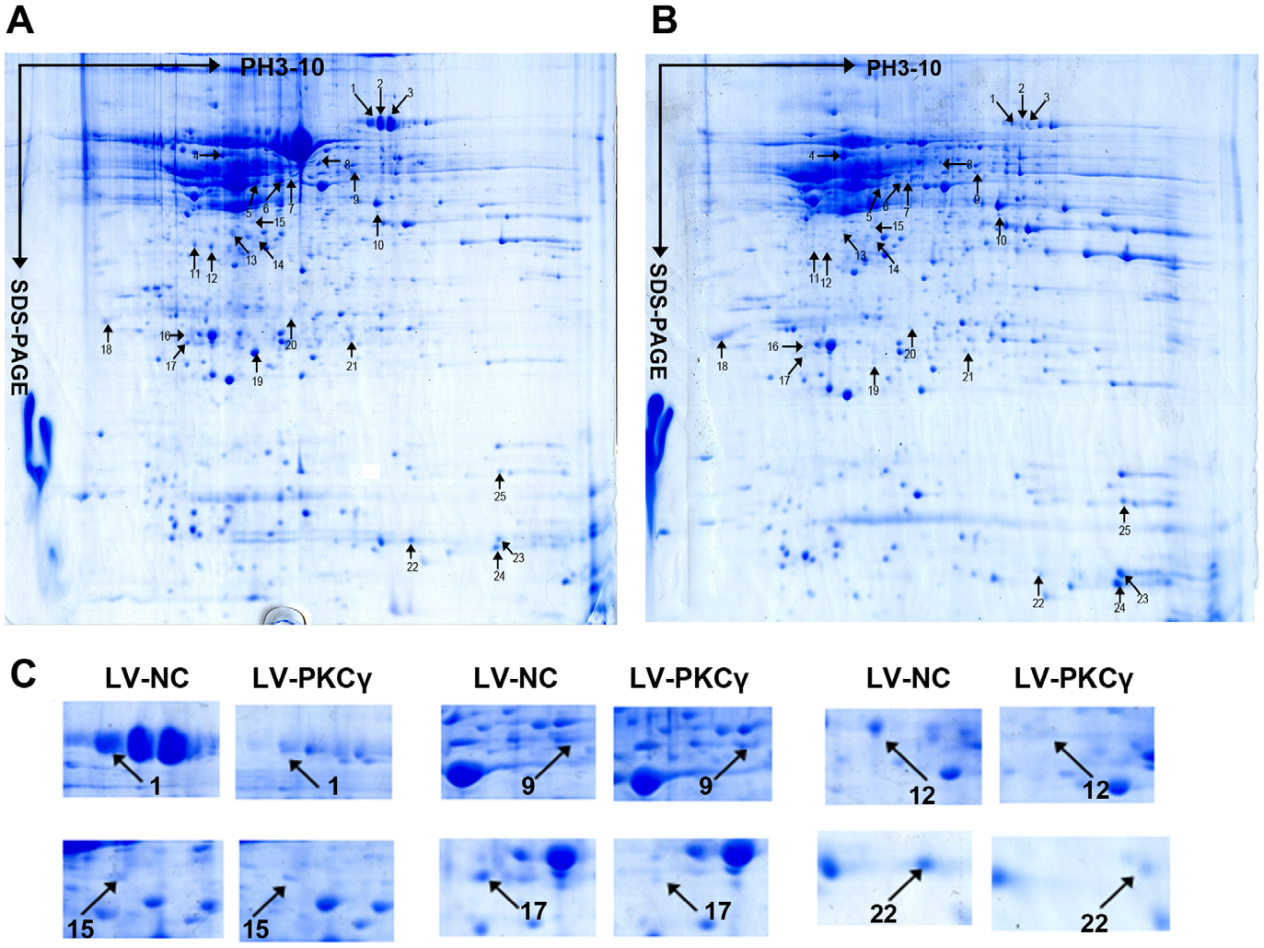
2DE images of the intumescentia lumbaris from the spinal cords of LV-NC- (A) or LV-shPKCã- (B) treated morphine-tolerant rats. Proteins were separated on a pH 4-7 IPG strip in the first dimension, and on an SDS-polyacrylamide (12%) gel in the second dimension. (C) Close-up image of partial differential expression protein spots, between LV-NC and LV-shPKCã.

To identify the proteins associated with the role of PKCã in morphine tolerance, we initiated a comparative proteomic study of MT rats treated with LV-NC or LV-shPKCã. Spinal cord proteins of rats were solubilized and separated using IPG strips and SDS-PAGE. Following staining with colloidal coomassie blue, well-resolved and reproducible 2D gel maps of the spinal cord were obtained, as shown in Figure 3A and 3B. Using PDQuest 2DE gel analysis software, approximately 1100 well-stained, clearly-delineated protein spots were detected, and most of these were distributed in the pH range from pH 5 to pH 8. Twenty-five spots were observed that showed significant changes between the LV-NC and LV-shPKCã groups, of which 7 were up-regulated and 18 down-regulated. These DEP spots are highlighted with arrows in Figure 3A and 3B. A close-up of the region of the gel showing DEP is presented in Figure 3C.

**Figure 4:**
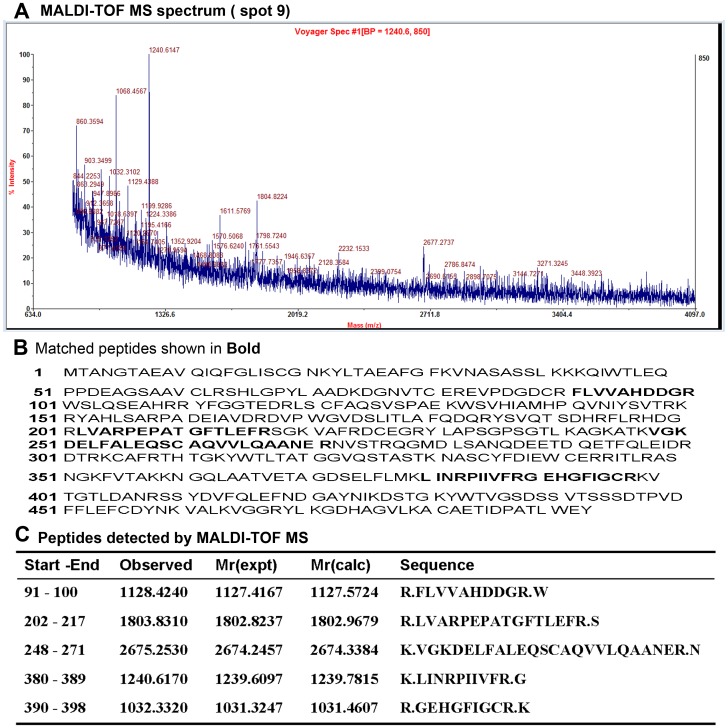
The PMF spectrum obtained using MALDI-TOF MS after tryptic digestion of a representative protein spot (spot 9).

DEP spots were excised from the 2DE gel, and MALDI-TOF MS analysis carried out to allow protein identification. Mass spectrometry analysis resulted in the identification of 13 distinct proteins; of these, some protein spots (such as spots 1, 2 and 3) presented as trains of similar molecular weight but different pIs, indicative of post-translational modifications. Three proteins were significantly up-regulated (≥2-fold; *P*<0.05) in the LV-shPKCã group, including peptidyl-prolyl cis-trans isomerase A in spot 25, protein ADP-ribosylarginine hydrolase in spot 14, and hemoglobin subunit beta-1 in spot 23. Ten proteins were down-regulated (≥2-fold; *P*<0.05) in the LV-shPKCã group, including serotransferrin in spots 1, 2 and 3, alpha-internexin in spot 4, glial fibrillary acidic protein in spot 5, fascin in spot 9, haptoglobin in spot 12, glutaredoxin-3 in spot 15, Rho-related GTP-binding protein RhoB in spot 17, guanidinoacetate N-methyl transferase in spot 20, glial cell line-derived neurotrophic factor in spot 21, and protein S100-A9 in spot 22. Table 1 presents a complete list of the identified proteins, including details of the quantification and MS analysis. Literature-based bioinformatics on the Swiss-Prot protein annotation page demonstrated that these various expressed proteins are involved in a variety of biological functions, including roles in the cytoskeleton, in ion metabolism, in post-translational modifications, as redox enzymes and as neurotrophins. The MALDI-TOF MS spectrum, the detected peptides, and the matched protein in a representative spot (spot 9) are shown in Figure 4, and the other spots’ information are shown in Figure S1 (supplementary data).

**Table 1:** Differentially expressed proteins in the spinal cords of PKCã-knockdown morphine tolerant rats, identified with 2DGE-based comparative proteomics.

Spot No	Protein id	Accession number	Protein name	Coverage (%)	MOWSE score	pIa	MWa (Da)	Expressional fold change
Proteins involved in ion metabolism								
1	TRFE-RAT	P12346	Serotransferrin	23	169	7.14	78512	0.31↓
2	TRFE-RAT	P12346	Serotransferrin	37	274	7.14	78512	0.34↓
3	TRFE-RAT	P12346	Serotransferrin	30	215	7.14	78512	0.26↓
12	HPT-RAT	P06866	Haptoglobin	20	77	6.10	39052	0.33↓
22	S10A9-RAT	P50116	Protein S100-A9	32	80	7.05	13307	0.15↓
23	HBB1-RAT	P02091	Hemoglobin subunit beta-1	66	163	7.88	16083	2.00↑
Proteins involved in cytoskeleton								
4	AINX-RAT	P23565	Alpha-internexin	52	196	5.20	56253	0.24↓
5	GFAP-RAT	P47819	Glial fibrillary acidic protein	24	57	5.35	49984	0.43↓
9	FSCN1-RAT	P85845	Fascin (Fragments)	13	56	6.29	55198	0.16↓
17	RHOB-RAT	P62747	Rho-related GTP-binding protein RhoB	43	63	5.10	22565	0.33↓
Proteins involved in post translational modification								
14	ADPRH-RAT	Q02589	Protein ADP-ribosylarginine hydrolase	29	55	5.62	40220	2.43↑
20	GAMT-RAT	P10868	Guanidinoacetate N- methyltransferase	55	136	5.69	26675	0.15↓
25	PPIA-RAT	P10111	Peptidyl-prolyl cis-trans isomerase A	74	106	8.34	18091	2.21↑
Redox enzymes								
15	GLRX3-RAT	Q9JLZ1	Glutaredoxin-3	25	66	5.51	38111	0.39↓
Neurotrophin								
21	GDNF-RAT	Q07731	Glial cell line-derived neurotrophic factor	26	53	9.04	24060	0.15↓

Up-arrows  =  up-regulated. Down-arrows  =  down-regulated. The % coverage of the analyzed peptides, the score from Mascot searches, the mass and pI (from the database) and Swiss-Prot accession number are shown for each protein. Proteins displaying up-regulation or down-regulation, with an average fold-difference (*P*<0.05) of ≥2-fold between pairs of conditions, are marked (+) and (−), respectively. MALDI-TOF MS result for % coverage of analyzed peptide and the score from Mascot searches.

To confirm the reliability of our proteomic analysis, we randomly selected three proteins for Western blot analysis: glial fibrillary acidic protein (GFAP), fascin (FSCN1), and glial cell line-derived neurotrophic factor (GDNF). As shown in Figure 2, the results from Western blot experiments were identical to the 2DGE analysis, comparing LV-shPKCã rats to controls.

### Effects of Chronic Morphine Administration and LV-shPKCã Treatment on Fascin Expression in the Spinal Cord, as Determined by Immunohistochemistry

**Figure 5:**
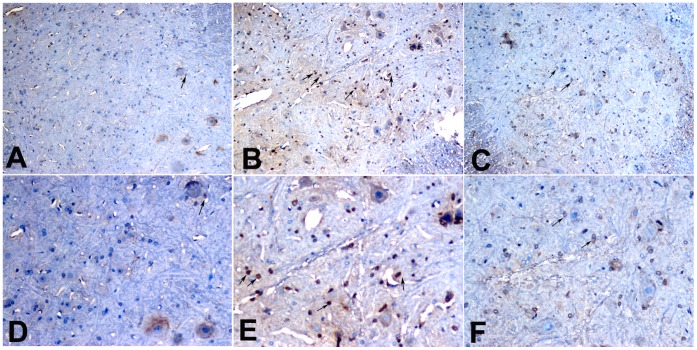
Immunohistochemical analysis of fascin expression in the spinal cord. Fascin staining was more intense in the chronic morphine exposure group than in the control group (A, D). In the LV-shPKCã group (C, F), fascin staining was significantly attenuated compared with the LV-NC group (B, E). Magnification: A, B, C 100×; D, E, F 200×.

As shown in Figure 5, chronic morphine exposure up-regulated the expression of fascin in the spinal cord, with staining for fascin being more intense in the morphine-treated group than in the control group. In the LV-shPKCã group, fascin staining was significantly attenuated compared with the LV-NC group.

## Discussion

**Figure 6:**
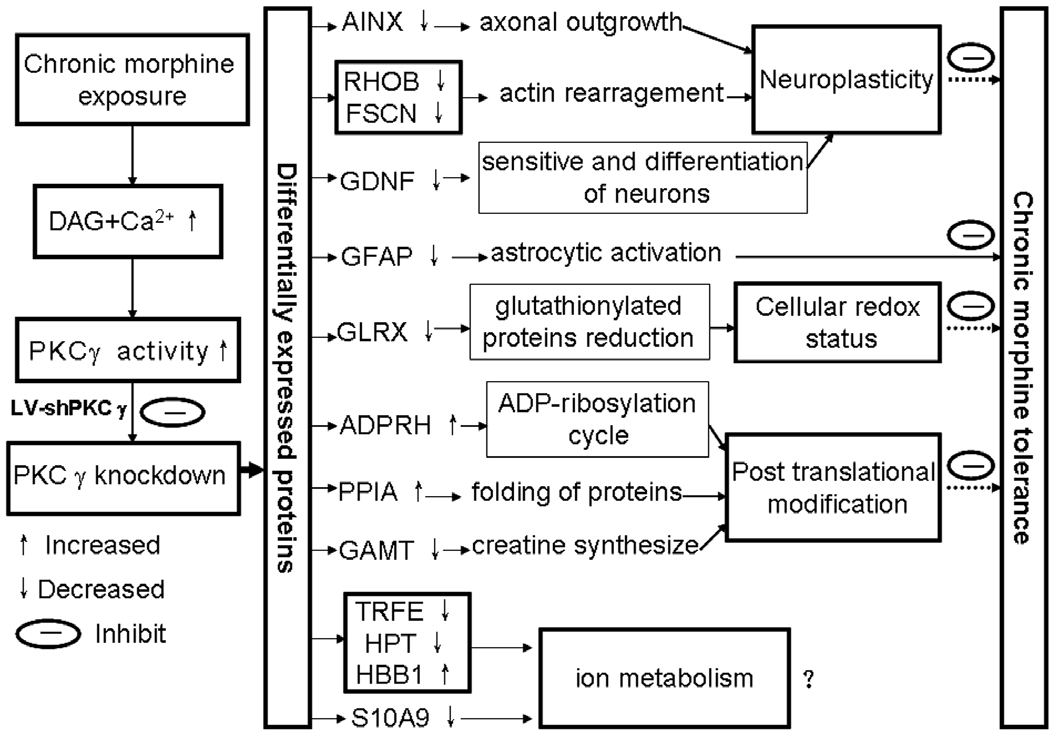
Experimental data-based diagram that rationalizes each DEP in the biological system that PKCã down-regulation inhibits morphine tolerance. AINX, alpha-internexin; RHOB, Rho-related GTP-binding protein RhoB; FSCN, fascin; GDNF, glial cell line-derived neurotrophic factor; GFAP, glial fibrillary acidic protein; GLRX, glutaredoxin; ADPRH, Protein ADP-ribosylarginine hydrolase; PPIA, peptidyl-prolyl cis-trans isomerase A; GAMT, guanidinoacetate N-methyltransferase; TRFE, serotransferrin; HPT, haptoglobin; HBB1, hemoglobin subunit beta-1; S10A9, protein S100-A9.

In the present study, we have combined a proteomic approach with RNAi technology to identify proteins associated with the role of PKCã in MT rats. To the best of our knowledge, this represents the first use of proteomics in combination with RNAi to investigate novel targets of PKCã in morphine tolerance. Our study identified 7 differential gel-spots in which spot volume increased, and 18 spots in which spot volume decreased. Among these, 13 DEPs were identified, using MALDI-TOF MS, whose expression differed between MT rats with PKCã knockdown and those without. These DEPs participate in a variety of cellular functions, including roles in the cytoskeleton, as neurotrophic factors, in oxidative stress, in ion metabolism, in cell signaling, and as chaperones. It has been well documented that opioid chronic exposure resulted in a large range of neuroadaptations at different levels of organization in the nervous system, such as opioid receptor itself, cellular, synaptic and system adaptations [30], [31], [32], [33]. Opioid tolerance results from adaptations due to homeostatic mechanisms tend to restore normal function in spite of the continued perturbations produced by opioid agonists. In the present study, we observed many adaptations related to chronic morphine administration and PKCã knockdown including neuroplasticity, astrocytic activation, post translational modificaion and changes in cellular redox status (Figure 6).

### Proteins Involved in the Cytoskeleton

In keeping with other findings, we observed changes in the expression of cytoskeletal proteins in the present study [34]. We found that intermediate filaments (IFs) and fascin were down-regulated after reversal of morphine tolerance by LV-shPKCã. IFs are 8∼10 nm structures that, together with microtubules (24∼26 nm) and microfilaments (6∼8 nm), form the cytoskeleton that is present in nearly all eukaryotic cells. In the CNS, the primary components of neuronal IFs are alpha-internexin (AINX) and the neurofilament triplet proteins. The major IF protein in astrocytes is glial fibrillary acidic protein (GFAP), although there are also lower levels of other IFs.

Increased expression of GFAP is commonly referred to as astrocytic activation [34], [35]. The activation of spinal glia may mediate and/or modulate opioid actions, such as morphine analgesia, tolerance and withdrawal. Growing body of studies focus on the role of glia in morphine tolerance, and find that activated glia is a rich source of cytokines that can profoundly influence neural activity and synaptic plasticity [36] **.** Although Song and Zhao have determined that co-administration of morphine with the glial metabolic inhibitor, fluorocitrate, significantly attenuated both astrocytic activation and tolerance to morphine analgesia, the role of glia in opioid tolerance is still unclear [35]. Brodie and colleagues have demonstrated differing roles for specific PKC isoforms in the proliferation of glial cells and the expression of the astrocytic marker, GFAP, and have observed that cells expressing PKCã displayed a marked increase in the levels of GFAP [37]. Furthermore, Narita et al. found a dramatic increase in the number of reactive astrocytes in the dorsal horn of the spinal cord following repeated treatment with morphine, whereas mice lacking PKCã failed to show astroglia hypertrophy or proliferation after morphine administration [16]. In the present study, expression of GFAP in the LV-shPKCã group was significantly down-regulated, compared with the control group, which is consistent with this previous report. Taken together, we can infer that activation of neuronal PKCã is implicated in the increased levels of reactive astrocytes following chronic treatment with morphine.

Fascin was first isolated from sea urchin egg, and was subdivided into 3 isoforms (fascin 1, 2 and 3). Initially, fascin was characterized as a 55-kDa actin cross-linking protein, expressed by normal mesenchymal, neuronal and dendritic cells, that contributes to the assembly of cortical cell protrusions and filopodia [38]. Subsequently, studies on cancers reported that fascin binds not only to actin, but also to a number of other proteins that can regulate cellular function. Fascin contains N- and C-terminal actin-binding sites; PKC binds to and phosphorylates fascin at serine-39 within the N-terminal actin-binding domain, resulting in the loss of actin bundling by fascin [39], [40]. Studies on carcinoma cells have confirmed that fascin is a substrate of PKC, and that a phosphomimetic form of fascin interacts preferentially with active PKCã [41]. It has also been documented that fascin is highly expressed in neurons, and participates in neuronal growth cone morphogenesis and reorganization [42], [43]. Furthermore, Kim and coworkers have reported the phosphorylation of fascin in rat brain after chronic morphine treatment [44]. In view of the above studies, we paid particular attention to observing the role of fascin and PKCã in morphine tolerance. The results of our immunohistochemistry experiments revealed that chronic administration of morphine induced anti-nociceptive tolerance to the drug, and up-regulated the expression of fascin in the spinal cord; in contrast, LV-shPKCã reversed morphine tolerance and down-regulated fascin expression. Our findings suggest that fascin contributes to the development of morphine tolerance in the spinal cord, and that the interaction between fascin and PKCã may play a key role in the development and maintenance of morphine tolerance. Rho-related GTP-binding protein is a member of the Ras superfamily of GTPases. In mammals, Rho GTPases are highly conserved and comprise Rho (A to H isoforms), Rac (Rac1 and Rac2 isoforms), Cdc42 (Cdc42Hs and G25K isoforms), and more distant members. The activation of Rho proteins mainly results in a rearrangement of the actin-based cytoskeleton and alterations in phosphoinositide levels [45]. Plenty of studies approved that Rac and Cdc42 are upstream regulators of the organisation of fascin and F-actin [41], [46], [47]. Rho proteins are thought to play an important role in cell proliferation, apoptosis, and gene expression, as well as carry out multiple other cellular functions [48].

### Neurotrophin

Glial cell line-derived neurotrophic factor (GDNF) is a distantly-related member of the transforming growth factor-beta superfamily,and thought to play an important role in the development and maintenance of several neurotransmitter systems, including the noradrenergic, GABAergic, and dopaminergic systems [49]. It has been well documented that GDNF is crucial for the neuroplastic changes related to chronic morphine administration [50], [51]. Chronic morphine induces a reduction in the size of dopamine neurons ventral tegmental area (VTA), this structural plasticity can be prevented by intra-VTA infusion of BDNF [52]. Repeated treatment with morphine for 9 days has been observed to increase GDNF levels in the nucleus accumbens, compared with levels in saline-treated mice [53]. However, little is known about alterations in GDNF in the spinal cord after morphine administration. A study in human astrocytes in cell culture observed that exposure to PKC inhibitors resulted in a dramatic down-regulation of GDNF mRNA [54]. With regard to a potential role in morphine tolerance, there is little information available in the literature concerning an interaction between PKCã and GDNF. Therefore, further studies are needed to investigate any changes in GDNF associated with chronic morphine treatment and PKCã knockdown.

### Proteins Involved in Ion Metabolism

Our study also observed alterations in proteins involved in iron metabolism, after reversal of morphine tolerance by LV-shPKCã. Hemoglobin is the iron-containing oxygen-transport protein found in red blood cells. A recent study has suggested that hemoglobin may act as an antioxidant and regulator of iron metabolism in the brain [55]. Haptoglobin binds free hemoglobin and thereby inhibits its oxidative activity. Transferrin is a glycoprotein that binds iron very tightly but reversibly, and transports it into the cell. The transferrin receptor is often considered as a marker of membrane recycling and of the early endocytic pathway. Studies of membrane recycling of transferrin have shown that PKC mediates transmembrane signals and regulates recycling endosomes, and that a PKC activator could accelerate endocytosis in a concentration-dependent manner [56]. In addition, Gomez and Weber reported that acute morphine injection significantly down-regulated the expression of transferrin receptors [57].

S100 proteins comprise a family of low molecular weight, EF-hand calcium-binding proteins with approximately 20 members [58]. Protein S100-A9 belongs to the calgranulin B subgroup of the S100 family, and is mainly associated with acute/chronic inflammatory disorders and various cancers [59]. Studies of opioid abuse have shown that morphine treatment resulted in diminished secretion of the antimicrobial proteins, S100-A9 and S100-A8/A9, in lung tissue and samples of bronchoalveolar lavage fluid during the early stages of Streptococcus pneumoniae infection [60]. It also has been reported that PKC may be involved in the stimulation of S100 synthesis and phosphorylation [61].

### Enzymes Involved in Redox Reactions and Post-translational Modifications

In the present study, we observed that various enzymes involved in post-translational modifications, redox reactions, and catalysis of chemical reactions, were altered after reversal of morphine tolerance by LV-shPKCã. Glutaretoxin uses the reducing power of glutathione to maintain and regulate the cellular redox state during oxidative stress [62]. It has been well documented that proteins related to oxidative stress may take contribute to the induction of morphine tolerance and dependence [63], [64].

In conclusion, proteomics in combination with RNAi is a useful method for the investigation of novel target molecules (in this study, PKCã) that may contribute to morphine tolerance. DEPs between MT models, with and without PKCã-knockdown, were identified with 2DGE-based comparative proteomics. These DEPs subserve a variety of functions, including roles in the cytoskeleton, as neurotrophic factors, in oxidative stress, in ion metabolism, in cell signaling, and as chaperones. These proteins, and in particular fascin, may prove to be potential molecular targets for future clinical treatment of morphine tolerance.

Based upon our experimental data, Figure 6 attempts to rationalize the role of each DEP in the mechanism by which PKCã down-regulation inhibits morphine tolerance. Our findings provide new clues that may help clarify the molecular mechanisms by which PKCã regulates neuropathic pain.

### Strengths and Limitations

The current study represents the first demonstration of the use of proteomics in combination with RNAi to investigate novel target molecules that may be involved in mediating the effects of PKCã on morphine tolerance. Thirteen DEPs were identified between MT rats, with and without PKCã knockdown (Table 1), and their roles in various biological systems assessed with regard to the possible mechanisms by which PKCã down-regulation inhibits morphine tolerance (Figure 6). Furthermore, three interesting DEPs (GFAP, FSCN and GDNF) were validated with Western blot and/or immunohistochemistry techniques. These findings enrich our understanding of how PKCã regulates morphine tolerance, thus achieving the first goal in our long-term program to investigate the biological role of PKCã in morphine tolerance, and develop potential clinical applications. However, additional experimental studies are needed to further clarify the involvement of these DEPs in the effects of PKCã on morphine tolerance, and to discover novel therapeutic targets for treating morphine tolerance.

## Materials and Methods

### Construction of Lentiviral Vectors Expressing PKCã shRNA

The siRNA system was synthesized as described in our previous studies [20], [65], [66]. Lentiviral vectors pGCSIL-GFP-shRNA were generated using sense siRNA sequences targeting PKCγ (GenBank accession number 012628) or negative control sequences (LV-NC): siPKCγ (1,343–1361): CTCTATGCCATCAAGATAC; nonsilencing siRNA: TTCTCCGAACGTGTCACGT. The oligonucleotides were designed according to the structure of the siRNA sense strand-loop-siRNA antisense strand. The shRNAs were cloned into lentiviral vector pGCSIL-GFP(Shanghai Gene Chemical Co., Ltd, Shanghai, China).To generate the lentivirus, the recombinant vector and packaged plasmids were cotransduced into 293T cells using lipofectamine 2000 (Invitrogen, Carlsbad, CA, USA). The final titer of recombinant virus was 1×10^9^ TU/ml.

### Animals and Treatments

A total of 42 male Sprague-Dawley rats, weighting 300-350 g, were used. Rats were housed in individual cages, with water and food pellets available ad libitum, and kept at 25°C with a 12-h light/dark cycle. The study was reviewed and approved by the Animal Care and Use Committee of Xiangya Medical College of Central South University, and adhered to the Ethical Guidelines of the International Association for the Study of Pain.

Rats were implanted with an intrathecal polyethylene-0402 catheter, inserted through an incision in the atlanto-occipital membrane into the subarachnoid space of the spinal cord, at the level of the rostral lumbar enlargement segments, according to a method described previously [20]. Rats that showed post-operative neurological deficits were excluded from the study. Animals were allowed to recover for 3 days before use in the experiments.

### Morphine Treatment and Behavioral Testing

As in previous studies, morphine tolerance was induced by an intrathecal treatment regimen: 10 µg of morphine was given twice daily for 6 consecutive days. On day 7, rats were injected intrathecally with LV-NC or LV-shPKCã.

A paw stimulation test was used to assess nociception, and was performed by an investigator blinded to the solution administered to each animal. The latencies of the hind-limb withdrawal response to radiant heat were measured prior to morphine injection, and 30 min after each morphine injection. To assess the thermal nociception threshold, rats were placed on the glass surface of a thermal testing apparatus (Ugo, Basile, Italy) and allowed to acclimate for 30 min before testing. The temperature of the glass surface was maintained constant at 30°C. A mobile radiant heat source located under the glass was focused onto the hind paw. The paw withdrawal latency was defined as the time that elapsed from the onset of radiant heat stimulation to withdrawal of the rat’s hind paw. Measurements were repeated 3 times in each rat, separated by a 3-min inter-trial interval, on both the left and right paws. A cut-off time of 20 s was used to prevent thermal injury.

### Sample Preparation

On day 14, twelve rats in the LV-NC and LV-shPKCã groups were killed, and the lumber enlargement (intumescentia lumbaris) of the spinal cord removed. The tissues were homogenized in 1 mL lysis buffer (7 M urea, 2 M thiourea, 100 mM DTT, 0.5 mM EDTA, 40 mM Tris, 2% NP40, 1% Triton X-100, 5 mM PMSF, and 2% Pharmalyte) for 2 h. The homogenate was centrifuged at 12000 rpm for 45 min at 4°C to remove tissue. After measurement of protein content using the BCA assay method (Pierce, Rockford, IL, USA), the products were stored at −80°C until further use. In each group, 6 samples were used for Western blot analysis, and 6 samples were used for two-dimensional gel electrophoresis (2DGE).

### Western Blot Analysis

Whole protein extracts (50 µg) from the spinal cords of the rats were subjected to 8% SDS-PAGE. After electrophoresis, the proteins were transferred to a PVDF membrane, blocked with a 5% nonfat dry milk for 1 h, and then incubated with rabbit anti-PKCã antibody (1∶200, Santa Cruz, CA, USA), rabbit anti-GFAP antibody (1∶1000, Santa Cruz), rabbit anti-GDNF antibody (1∶1000, Santa Cruz), or rabbit anti-FSCN1 antibody (1∶1000, Santa Cruz), for 1.5 h at 25°C. The membranes were washed three times (10 min each) in Tris-buffered saline containing 0.1% Tween-20, and the secondary antibody (1∶5000 dilution of horseradish peroxidase-coupled goat anti-rabbit immunoglobulin G; Sigma, St Louis, MO, USA) was added for 1.5 h at 25°C. â-actin was detected simultaneously with a mouse anti-actin monoclonal antibody (1∶5000, Sigma), as a loading control. The membranes were washed in washing buffer for a further 30 min, and the antibodies were then revealed using Super Signal® West Pico Chemiluminescent Substrate (Pierce, Rockford, IL, USA), with the blot exposed to X-ray film. Band density was measured using Quantity One analysis software (Bio-Rad, Hercules, CA, USA), and the results were expressed as the ratio of immunoreactivity of the protein of interest to â-actin immunoreactivity.

### Two-dimensional Gel Electrophoresis (2-DE)

Total proteins from 6 different rats of each group were analyzd separately. Both samples of spinal cords were carried out 2-DE in parallel. 2-DE was performed as described with minor modifications [67]. IPG strips were used according to the manufacturer’s instruction. The amounts of proteins (450 µg) were mixed with an aliquot (450 µl) of rehydration solution (8 M urea, 40 mM Tris, 4% CHAPS, 18 mM DTT, a trace of bromophenol blue, and 0.5% IPG buffer pH 4-7; Amersham Pharmacia Biotech, Piscataway, NJ) then applied on IPG strips. Isoelectric focusing (IEF) was performed with the following voltage-time program: 30 V for 14 h, 500 V for 1 h,1000 V for 1 h, and 8000 V for 8.5 h, for a total of 120 000V/h. Prior to SDS-PAGE, IPG strips were equilibrated in a solution (6 M urea, 2% SDS, 0.2% DTT, 50 mM Tris, pH 8.8, 30% glycerol) for 15 min, followed by equilibration in a solution(6 M urea, 2% SDS, 3% iodoacetamide, 50 mM Tris, pH 8.8, 30% glycerol) for 15 min. The second dimension separation was run for 5 h using a vertical electrophoresis system (Amersham Pharmacia Biotech, Piscataway, NJ) in 0.75-mm 12% gels. The gels were stained with colloidal coomassie blue, and were then scanned with image scanner (Amersham Pharmacia Biotech, Piscataway, NJ).

Gel image analysis was done with PDQuest 8.01 software (Bio-Rad, USA). The student t-test was applied to compare the spot relative volume (% vol) in gels derived from individual LV-NC and LV-shPKCã treated animals. Spot intensities were quantified with spot-volume [O.D. x (I.U.)^2^]. Each spot-volume in a gel was normalized with the total spot-volume of this gel. The normalized spot-volume was used to determine each DEP with a difference ≥2-fold variation in group LV-shPKCã compared to group LV-NC.

### In-gel Tryptic Digestion and Protein Identification by Mass Spectrometry

Protein spots were excised from the preparative gels and transferred into 1.5 mL eppendorf tubes. The gel pieces were destained in 50% acetonitrile (ACN) in 50 mM ammonium bicarbonate buffer, and dehydrated with 100% ACN. The gel pieces were then re-hydrated in 10 µL trypsin solution (100 mg/mL) for 40 min. After incubation overnight at 37°C, the peptides were extracted two times with 30 µL 5% trifluoroacetic acid (TFA) in 100% ACN. Extracts were pooled together and lyophilized. 0.5 µL incubation buffer was mixed with 1 µL matrix solution (CHCA, 2 mg/mL in 50% ACN and 1% TFA), and this was pipetted directly onto the stainless steel sample plate of the mass spectrometer.

The samples were analyzed using a Voyager-DE STR 4307 MALDI-TOF mass spectrometer (ABI Applied Biosystem, Framingham, USA), according to previously described procedures [68]. The tryptic peptides were mixed with matrix solution containing α-cyano-4-hydroxycinnamic (CCA) (Sigma-Aldrich Co.), and the mixture (1 µL) was spotted onto the MALDI plate, and analyzed with the Voyager System mass spectrometer (MS) to obtain the MS spectrum for peptide mass fingerprint (PMF) analysis. The PMF data were used to identify proteins, using the Mascot search engine (http://www.matrixscience.com/) against the Swiss-Prot database. The parameters were set as: monoisotopic peak; mass tolerance 50 ppm; trypsin; and rat database. The coverage of the amino acid sequence, the Mascot score, the relative mass and pI generated from gels, the calculated mass and pI generated from the database, and Swiss-Prot accession number were obtained for each protein.

### Immunohistochemical Detection of Fascin

Rats were randomly divided into three groups as follows: control group (n = 6), LV-NC group (n = 6) and LV-shPKCã group (n = 6). In the control group, saline was administered intrathecally twice daily for 7 days. In the LV-NC and LV-shPKCã groups, rats were treated as described above. On day 13, rats in all three groups were anesthetized with chloral hydrate (500 mg/kg) and transcardially perfused with 4% buffered formalin following a normal saline flush. The spinal cords were removed and embedded in paraffin. Following dewaxing, dehydration, and antigen retrieval, sections (5 µm thick) were incubated overnight at 4°C with anti-fascin antibody. The samples were then incubated for 30 min with biotinylated secondary antibody, and subsequently treated for 30 min with a biotin–streptavidin complex. Sections were then stained with 3,3′-diaminobenzidine (DAB, Sigma, USA) for 10 min, washed with tap water, and counterstained with hematoxylin. Photographs were taken with an Olympus digital camera attached to a microscope equipped with 100× and 200× objectives.

### Statistical Analysis

All data were tested for normality before statistical analysis, which was carried out using SPSS13.0 software. All data are expressed as the mean ± SD. Student’s t-test was used for statistical comparisons. A value of *P*<0.05 was considered to be indicative of statistical significance.

## Supporting Information

Figure S1 **The PMF spectrums obtained using MALDI-TOF MS after tryptic digestion of thirteen protein spots.**
(PDF)
