# Supplementary material for: Proteomic Analysis of PKCγ-Related Proteins in the Spinal Cord of Morphine-Tolerant Rats
Source: PLoS One. 2012 Jul 31;7(7):e42068. doi: 10.1371/journal.pone.0042068 (PMC3409149; doi:10.1371/journal.pone.0042068)
Supplement: Figure S1 — The PMF spectrums obtained using MALDI-TOF MS after tryptic digestion of thirteen protein spots. (PDF) [file pone.0042068.s001.pdf]

A. MALDI-TOF MS spectrum (spot 1)

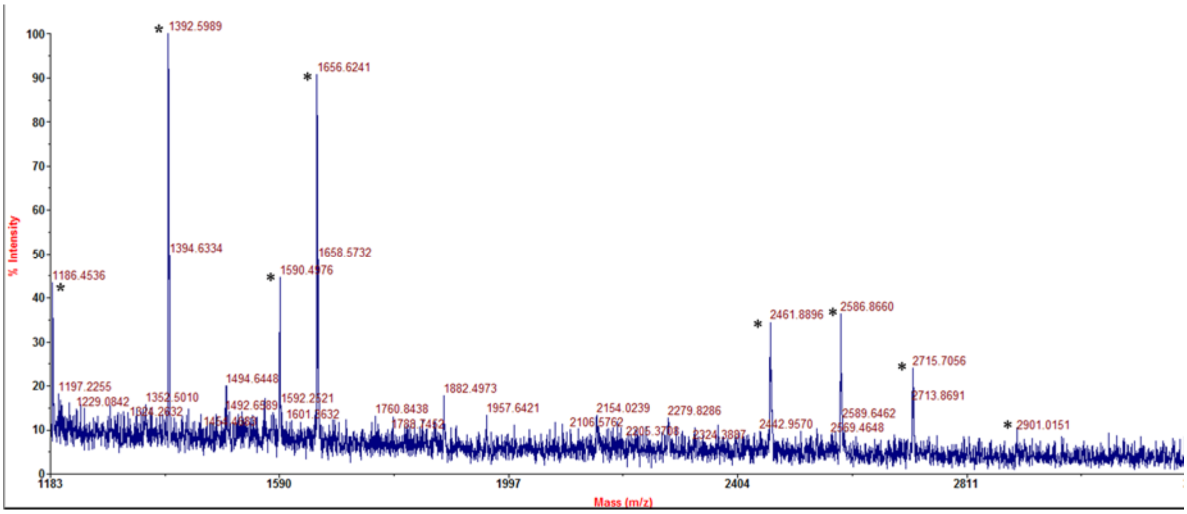

B. Peptides detected by MALDI-TOF-MS

Peptide	Amino acid sequence	$[M+H]^+$	Matched
144 - 162	SAGWIIPIGLLFCNLPEPR	2152.8170	+
237 - 251	ADRDQYELLCLDNTR	1881.5440	+
252 - 264	KPVDQYEDCYLAR	1656.5730	+
311 - 323	DLLFKDSAFGLLR	1494.6630	+
316 - 323	DSAFGLLR	878.3600	+
332 - 343	LYLGHSYVTAIR	1392.5980	+
362 - 370	WCALSHQER	1186.4070	+
478 - 491	TAGWNIPMGLLFSR	1562.5600	+
588 - 609	KPVTEFATCHLAQAPNHVVVSR	2460.8360	+
630 - 642	GDKDCTGNFCLFR	1589.4930	+
647 - 655	DLLFRDDTK	1122.4230	+
660 - 682	LPEGTTYEEYLGAEYLQAVGNIR	2585.9000	+
660 - 683	LPEGTTYEEYLGAEYLQAVGNIRK	2713.9180	+

C. Matched peptides shown in Bold Red

1 MRFAVGALLA CAALGLCLAV PDKTVKWCAV SEHENTKCIS FRDHMKTVLP
51 ADGPRLACVK KTSYQDCIKA ISGGEADAIT LDGGWVYDAG LTPNNLKPVA
101 AEFYGSLEHP QTHYLAVAVV KKGTDFQLNQ LQGKKSCHTG LGR**SAGWIIP**
151 **IGLLFCNLPE PR**KPLEKAVA SFFSGSCVPC ADPVAFPQLC QLCPGCGCSP
201 TQPFFGYVGA FKCLRDGGGD VAFVKHTTIF EVLPQK**ADRD QYELLCLDNT**
251 **RKPVDQYEDC YLAR**IPSHAV VARNGDGKED LIWEILKVAQ EHFGKGKSKD
301 FQLFGSPLGK **DLLFKDSAFG LLR**VPPRMDY R**LYLGHSYVT AIR**NQREGVC
351 PEGSIDSAPV K**WCALSHQER** AKCDEWSVSS NGQIECESAE STEDCIDKIV
401 NGEADAMSLD GGHAYIAGQC GLVPVMAENY DISSCTNPQS DVFPKGYYAV
451 AVVKASDSSI NWNNLKGKKS CHTGVDR**TAG WNIPMGLLFS R**INHCKFDEF
501 FSQGCAPGYK KNSTLCDLCI GPAKCAPNNR EGYNGYTGAF QCLVEKGDVA

551 FVKHQTVLEN TNGKNTAAWA KDLKQEDFQL LCPDGTK**KPV TEFATCHLAQ**
601 **APNHVVVSR**K EKAARVSTVL TAQKDLFWK**G DKDCTGNFCL FR**SSTK**DLLF**
651 **RDDTK**CLTK**L PEGTTYEEYL GAEYLQAVGN IRK**CSTSRLL EACTFHKS

A. MALDI-TOF MS spectrum (spot 2)

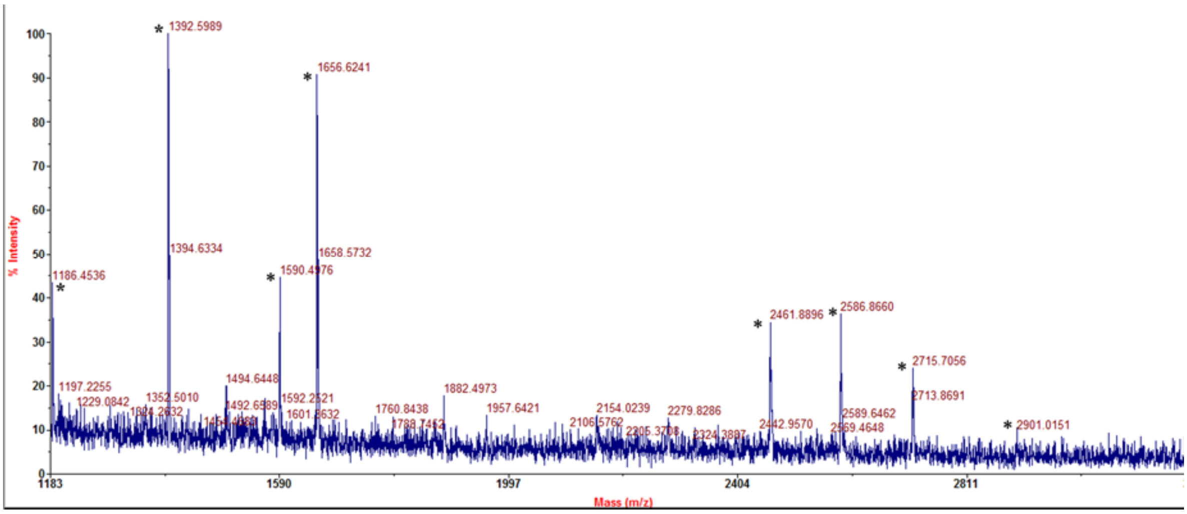

B. Peptides detected by MALDI-TOF-MS

Peptide	Amino acid sequence	$[M+H]^+$	Matched
27 - 37	WCAVSEHENTK	1360.6230	+
123 - 135	GTDFQLNQLQGKK	1476.7770	+
144 - 162	SAGWIIPIGLLFCNLPEPR	2153.1090	+
226 - 236	HTTIFEVLPQK	1312.7130	+
237 - 251	ADRDQYELLCLDNTR	1881.8200	+
240 - 251	DQYELLCLDNTR	1539.7160	+
252 - 264	KPVDQYEDCYLAR	1656.7540	+
265 – 273	IPSHAVVAR	949.5480	+
274 - 287	NGDGKEDLIWEILK	1629.8140	+
298 - 310	SKDFQLFGSPLGK	1423.7740	+
311 - 323	DLLFKDSAFGLLR	1494.8260	+
316 – 323	DSAFGLLR	878.4670	+
332 - 343	LYLGHSYVTAIR	1392.7660	+
362 - 370	WCALSHQER	1186.5410	+
455 - 466	ASDSSINWNNLK	1348.6510	+
478 - 491	TAGWNIPMGLLFSR	1562.8140	+
572 - 587	DLKQEDFQLLCPDGTK	1906.8800	+
588 - 609	KPVTEFATCHLAQAPNHVVVSR	2461.1780	+
630 - 642	GDKDCTGNFCLFR	1589.6840	+
633 - 642	DCTGNFCLFR	1289.5840	+
647 - 655	DLLFRDDTK	1122.5750	+
660 - 682	LPEGTTYEEYLGAEYLQAVGNIR	2586.1700	+
660 - 683	LPEGTTYEEYLGAEYLQAVGNIRK	2714.2800	+

C. Matched peptides shown in Bold Red

1 MRFAVGALLA CAALGLCLAV PDKTVK**WCAV SEHENTK**CIS FRDHMKTVLP

51 ADGPRLACVK KTSYQDCIKA ISGGEADAIT LDGGWVYDAG LTPNNLKPVA

101 AEFYGSLEHP QTHYLAVAVV KK**GTDFQLNQ LQGKK**SCHTG LGR**SAGWIIP**

151 **IGLLFCNLPE PR**KPLEKAVA SFFSGSCVPC ADPVAFPQLC QLCPGCGCSP

201 TQPFFGYVGA FKCLRDGGGD VAFVK**HTTIF EVLPQKADRD QYELLCLDNT**

251 **RKPVDQYEDC YLARIPSHAV VARNGDGKED LIWEILK**VAQ EHFGKGK**SKD**

301 **FQLFGSPLGK DLLFKDSAFG LLR**VPPRMDY R**LYLGHSYVT AIR**NQREGVC

351 PEGSIDSAPV K**WCALSHQER** AKCDEWSVSS NGQIECESAE STEDCIDKIV

401 NGEADAMSLD GGHAYIAGQC GLVPVMAENY DISSCTNPQS DVFPKGYYAV

451 AVVK**ASDSSI NWNNLK**GKKS CHTGVDR**TAG WNIPMGLLFS R**INHCKFDEF

501 FSQGCAPGYK KNSTLCDLCI GPAKCAPNNR EGYNGYTGAF QCLVEKGDVA

551 FVKHQTVLEN TNGKNTAAWA K**DLKQEDFQL LCPDGTKKPV TEFATCHLAQ**

601 **APNHVVVSR**K EKAARVSTVL TAQKDLFWK**G DKDCTGNFCL FR**SSTK**DLLF**

651 **RDDTK**CLTK**L PEGTTYEEYL GAEYLQAVGN IRK**CSTSRLL EACTFHKS

A. MALDI-TOF MS spectrum (spot 3)

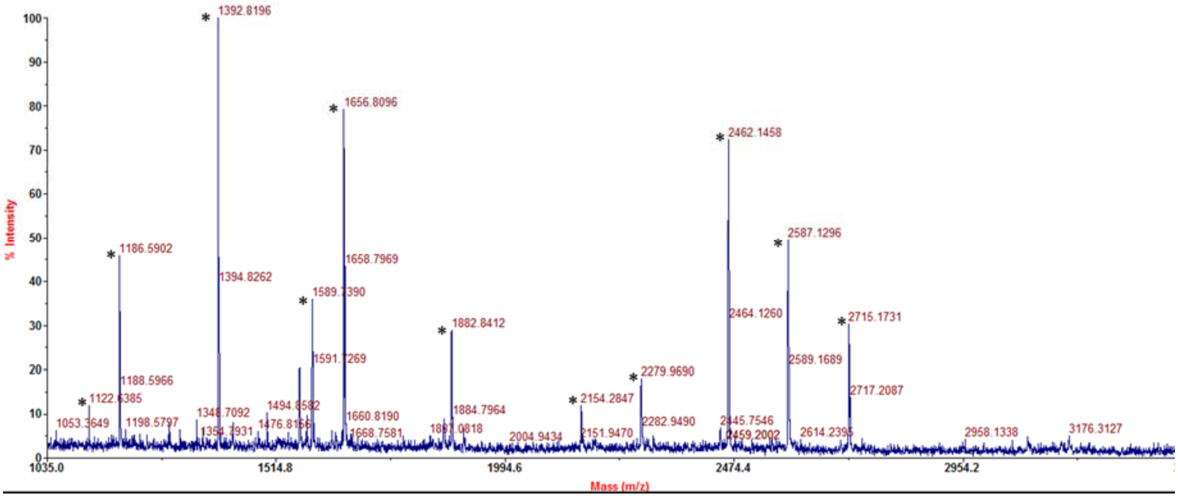

B. Peptides detected by MALDI-TOF-MS

Peptide	Amino acid sequence	$[M+H]^+$	Matched
123 - 134	GTDFQLNQLQGK	1348.7100	+
144 - 162	SAGWIIPIGLLFCNLPEPR	2153.1090	+
226 - 236	HTTIFEVLPQK	1312.7510	+
237 - 251	ADRDQYELLCLDNTR	1881.8410	+
252 - 264	KPVDQYEDCYLAR	1656.8090	+
298 - 310	SKDFQLFGSPLGK	1423.7940	+
311 - 323	DLLFKDSAFGLLR	1494.8580	+
316 - 323	DSAFGLLR	878.4940	+
332 - 343	LYLGHSYVTAIR	1392.6440	+
332 - 343	LYLGHSYVTAIR	1392.8180	+
362 - 370	WCALSHQER	1186.5890	+
478 -491	TAGWNIPMGLLFSR	1562.8720	+
478 - 491	TAGWNIPMGLLFSR	1578.8130	+
572 - 587	DLKQEDFQLLCPDGTK	1906.8430	+
588 - 609	KPVTEFATCHLAQAPNHVVVSR	2461.1530	+
630 - 642	GDKDCTGNFCLFR	1589.7300	+
633 –642	DCTGNFCLFR	1289.5940	+
647 - 655	DLLFRDDTK	1122.6310	+
660 - 682	LPEGTTYEEYLGAEYLQAVGNIR	2586.1390	+
660 - 683	LPEGTTYEEYLGAEYLQAVGNIRK	2714.1850	+

C. Matched peptides shown in **Bold Red**

1 MRFAVGALLA CAALGLCLAV PDKTVKWCAV SEHENTKCIS FRDHMKTVLP
51 ADGPRLACVK KTSYQDCIKA ISGGEADAIT LDGGWVYDAG LTPNNLKPVA
101 AEFYGSLEHP QTHYLAVAVV KK**GTDFQLNQ LQGK**KSCHTG LGR**SAGWIIP**
151 **IGLLFCNLPE PR**KPLEKAVA SFFSGSCVPC ADPVAFPQLC QLCPGCGCSP
201 TQPFFGYVGA FKCLRDGGGD VAFVK**HTTIF EVLPQKADRD QYELLCLDNT**

251 **RKPVDQYEDC YLAR**IPSHAV VARNGDGKED LIWEILKVAQ EHFGKGK**SKD**
301 **FQLFGSPLGK DLLFKDSAFG LLR**VPPRMDY R**LYLGHSYVT AIR**NQREGVC
351 PEGSIDSAPV K**WCALSHQER** AKCDEWSVSS NGQIECESAE STEDCIDKIV
401 NGEADAMSLD GGHAYIAGQC GLVPVMAENY DISSCTNPQS DVFPKGYYAV
451 AVVKASDSSI NWNNLKGKKS CHTGVDR**TAG WNIPMGLLFS R**INHCKFDEF
501 FSQGCAPGYK KNSTLCDLCI GPAKCAPNNR EGYNGYTGAF QCLVEKGDVA
551 FVKHQTVLEN TNGKNTAAWA K**DLKQEDFQL LCPDGTKKPV TEFATCHLAQ**
601 **APNHVVVSR**K EKAARVSTVL TAQKDLFWK**G DKDCTGNFCL FR**SSTK**DLLF**
651 **RDDTK**CLTK**L PEGTTYEEYL GAEYLQAVGN IRK**CSTSRLL EACTFHKS

A. MALDI-TOF MS spectrum (spot 4)

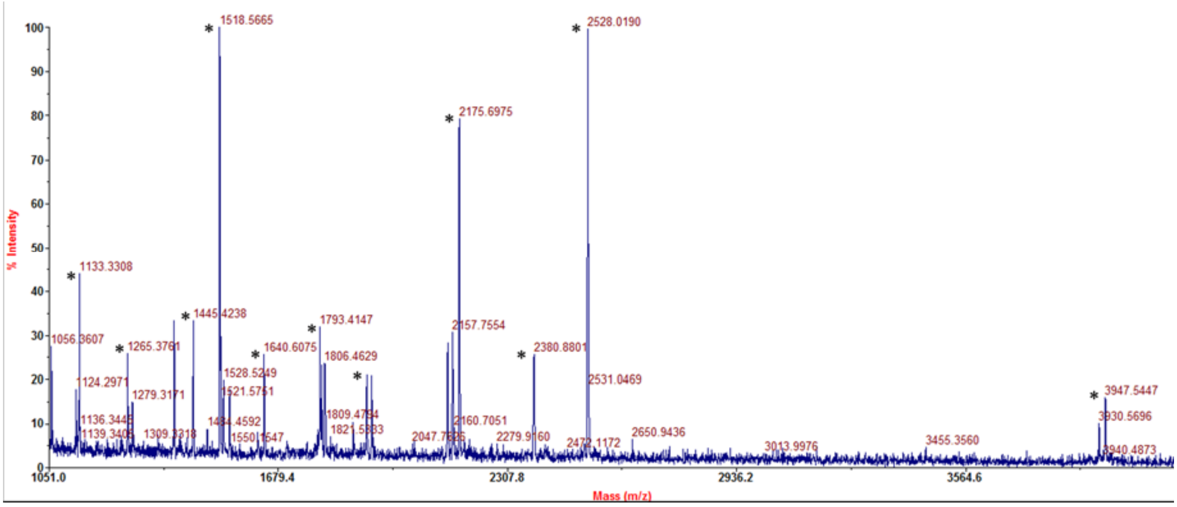

B. Peptides detected by MALDI-TOF-MS

Peptide	Amino acid sequence	$[M+H]^+$	Matched
46 - 67	SNVASTAACSSASSLGLGLAYR	2142.7180	+
68 - 83	RLPASDGLDLSQAAAR	1640.5580	+
69 - 83	LPASDGLDLSQAAAR	1484.4670	+
92 - 104	TNEKEQLQGLNDR	1544.4500	+
112 - 120	VHQLETQNR	1124.3020	+
121 - 130	ALEAELAALR	1056.3500	+
139 - 145	VGELFQR	848.2310	+
62 - 177	AQALLERDGLAEEVQR	1797.6320	+
216 - 228	KVESLLDELAFVR	1518.5740	+
229 - 266	QVHDEEVAELLATLQASSQAAAEVDVAVAKPDLTSALR	3945.5350	+
291 - 300	FANLNEQAAR	1133.3080	+
323 - 330	TIEIEGLR	930.2770	+
339 - 346	QILELEER	1029.3040	+
347 - 366	HSAEVAGYQDSIGQLESDLR	2174.6980	+
375 - 386	HLREYQDLLNVK	1527.5420	+
387 – 397	MALDIEIAAYR	1265.3820	+
387 - 398	MALDIEIAAYRK	1393.4450	+
399 - 430	LLEGEETRFSTSGLSISGLNPLPNPSYLLPPR	3454.3450	+
407 – 430	FSTSGLSISGLNPLPNPSYLLPPR	2527.0110	+
471 - 487	VGESFEETLEETVVSTK	1883.5440	+

C. Matched peptides shown in Bold Red

1 MSFGSEHYLC SASSYRKVFG DGSRLSARLS GPGASGSFRS QSLSR**SNVAS**

51 **TAACSSASSL GLGLAYRRLP ASDGLDLSQA AAR**TNEYKII R**TNEKEQLQG**

101 **LNDR**FAVFIE K**VHQLETQNR ALEAELAALR** QRHAEPSR**VG ELFQR**ELREL

151 RAQLEEASSA R**AQALLERDG LAEEVQR**LRA RCEEESRGRE GAERALKAQQ

201 RDVDGATLAR LDLEK**KVESL LDELAFVRQV HDEEVAELLA TLQASSQAAA**

251 **EVDVAVAKPD LTSALR**EIRA QYESLAAKNL QSAEEWYKSK **FANLNEQAAR**
301 STEAIRASRE EIHEYRRQLQ AR**TIEIEGLR** GANESLER**QI LELEERHSAE**
351 **VAGYQDSIGQ LESDLR**NTKS EMAR**HLREYQ DLLNVKMALD IEIAAYRKLL**
401 **EGEETRFSTS GLSISGLNPL PNPSYLLPPR** ILSSTTSKVS SAGLSLKKEE
451 EEEEEEEEGA SKEVTKKTSK **VGESFEETLE ETVVSTK**KTE KSTIEEITTS
501 SSQKM

A. MALDI-TOF MS spectrum (spot 5)

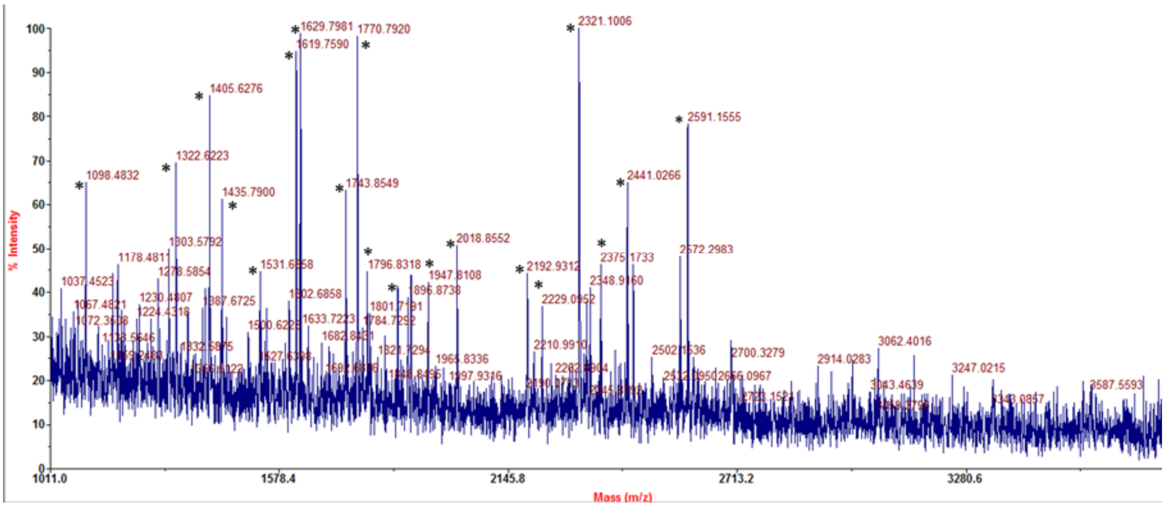

B. Peptides detected by MALDI-TOF-MS

Peptide	Amino acid sequence	$[M+H]^+$	Matched
69 - 84	AEMMELNDRFASYIEK	1946.8140	+
110 - 119	LADVYQAELR	1177.5040	+
135 - 150	LEVERDNLTQDLGTLR	1871.8600	+
188 - 200	VESLEEEIQFLRK	1619.7530	+
208 - 234	ELQEQLAQQQVHVEMDVAKPDLTAALR	3060.3720	+
367 - 388	LLEGEENRITIPVQTFSNLQIR	2570.2440	+
375 - 388	ITIPVQTFSNLQIR	1629.7980	+

C. Matched peptides shown in Bold Red

1 MERRRITSAR RSYASSETMV RGHGPTRHLG TIPRLSLSRM TPPLPARVDF
51 SLAGALNAGF KETRASER**AE MMELNDRFAS YIEK**VRFLEQ QNKALAAELN
101 QLRAKEPTK**L ADVYQAELR**E LRLRLDQLTT NSAR**LEVERD NLTQDLGTLR**
151 QKLQDETNLR LEAENNLAVY RQEADEATLA RVDLERK**VES LEEEIQFLRK**
201 IHEEEVR**ELQ EQLAQQQVHV EMDVAKPDLT AALR**EIRTQY EAVATSNMQE
251 TEEWYRSKFA DLTDVASRNA ELLRQAKHEA NDYRRQLQAL TCDLESLRGT
301 NESLERQMRE QEERHARESA SYQEALARLE EEGQSLKEEM ARHLQEYQDL
351 LNVKLALDIE IATYRK**LLEG EENRITIPVQ TFSNLQIR**ET SLDTKSVSEG
401 HLKRNIVVKT VEMRDGEVIK ESKQEHKDVM

A. MALDI-TOF MS spectrum (spot 9)

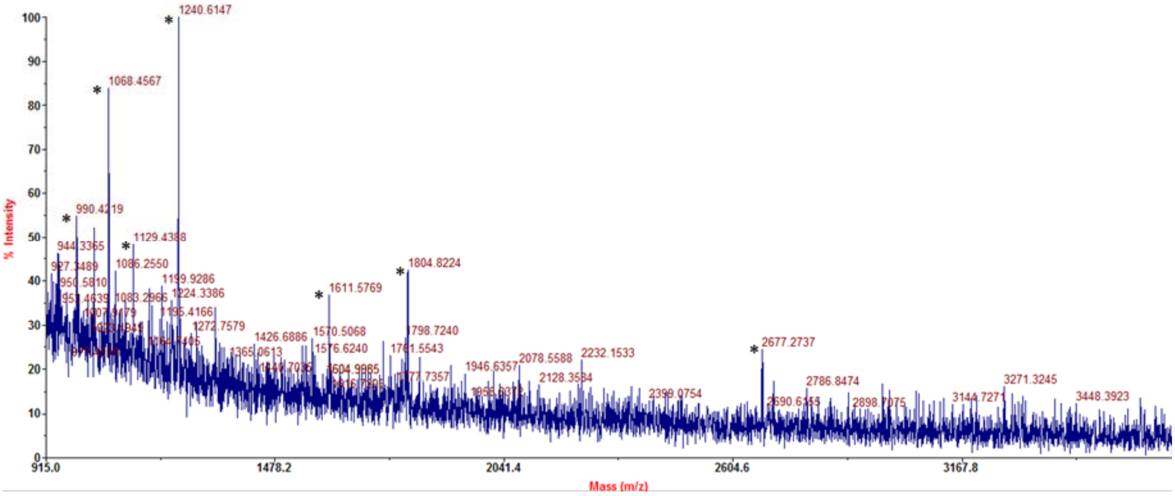

B. Peptides detected by MALDI-TOF-MS

Peptide	Amino acid sequence	$[M+H]^+$	Matched
91 - 100	FLVVAHDDGR	1128.4240	+
202 - 217	LVARPEPATGFTLEFR	1803.8310	+
248 - 271	VGKDELFALEQSCAQVVLQAANER	2675.2530	+
380 - 389	LINRPIIVFR	1240.6170	+
390 - 398	GEHGFIGCR	1032.3320	+

C. Matched peptides shown in Bold Red

1 MTANGTAEAV QIQFGLISCG NKYLTAEAFG FKVNASASSL KKKQIWTLEQ
51 PPDEAGSAAV CLRSHLGPYL AADKDGNVTC EREVPDGDCR **FLVVAHDDGR**
101 WSLQSEAHRR YFGGTEDRLS CFAQSVSPAE KWSVHIAMHP QVNIYSVTRK
151 RYAHLSARPA DEIAVDRDVP WGVDSLITLA FQDQRYSVQT SDHRFLRHDG
201 R**LVARPEPAT GFTLEFR**SGK VAFRDCEGRY LAPSGPSGTL KAGKATK**VGK**
251 **DELFALEQSC AQVVLQAANE R**NVSTRQGMD LSANQDEETD QETFQLEIDR
301 DTRKCAFRTH TGKYWTLTAT GGVQSTASTK NASCYFDIEW CERRITLRAS
351 NGKFVTAKKN GQLAATVETA GDSELFLMK**L INRPIIVFRG EHGFIGCR**KV
401 TGTLDANRSS YDVFQLEFND GAYNIKDSTG KYWTVGSDSS VTSSSDTPVD
451 FFLEFCDYNK VALKVGGRYL KGDHAGVLKA CAETIDPATL WEY

A. MALDI-TOF MS spectrum (spot 12)

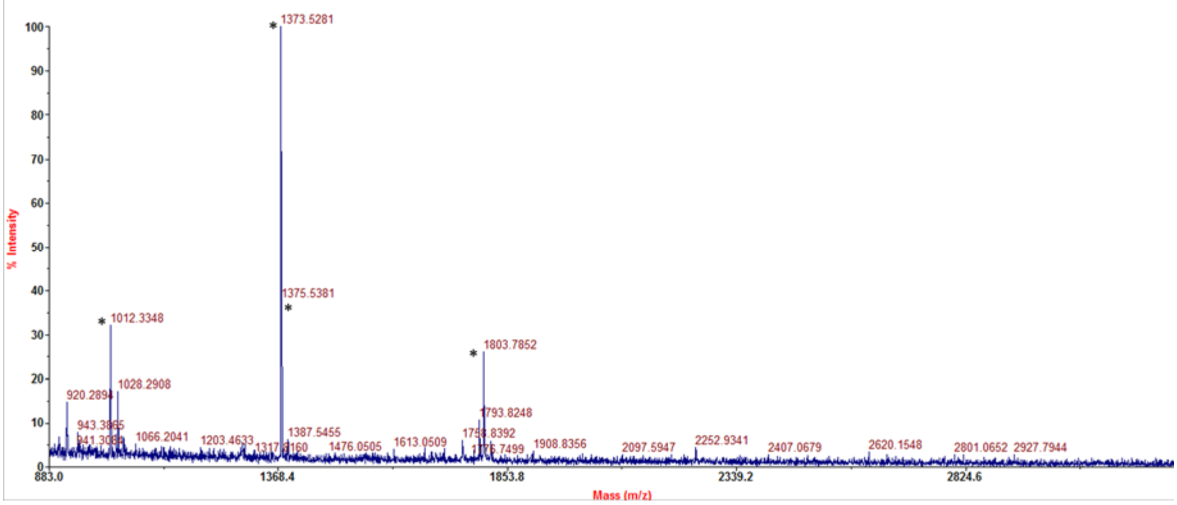

B. Peptides detected by MALDI-TOF-MS

Peptide	Amino acid sequence	$[M+H]^+$	Matched
112 - 119	GSFPWQAK	920.3050	+
177 - 183	VVLHPER	849.3150	+
203 - 218	VMPICLPSKDYVAPGR	1802.8090	+
203 - 218	VMPICLPSKDYVAPGR	1818.7930	+
219 - 227	MGYVSGWGR	1012.3610	+
219 - 227	MGYVSGWGR	1028.3020	+
239 - 256	YVMLPVADQEKCELHYEK	2251.9250	+
321 - 332	SCAVAEYGVYVR	1373.5550	+

C. Matched peptides shown in Bold Red

1 MRALGAVVTL LLWGQLFAVE LGNDATDIED DSCPKPPEIA NGYVEHLVRY
51 RCRQFYKLQT EGDGIYTLNS EKQWVNPAAG DKLPKCEAVC GKPKHPVDQV
101 QRIIGGSMDA K**GSFPWQAK**M ISRHGLTTGA TLISDQWLLT TAQNLFLNHS
151 ENATAKDIAP TLTLYVGKNQ LVEIEK**VVLH PER**SVVDIGL IKLKQKVLVT
201 EK**VMPICLPS KDYVAPGRMG YVSGWGR**NVN FRFTERLK**YV MLPVADQEKC**
251 **ELHYEK**STVP EKKGAVSPVG VQPILNKHTF CAGLTKYEED TCYGDAGSAF
301 AVHDTEEDTW YAAGILSFDK **SCAVAEYGVY VR**ATDLKDWV QETMAKN

A. MALDI-TOF MS spectrum (spot 14)

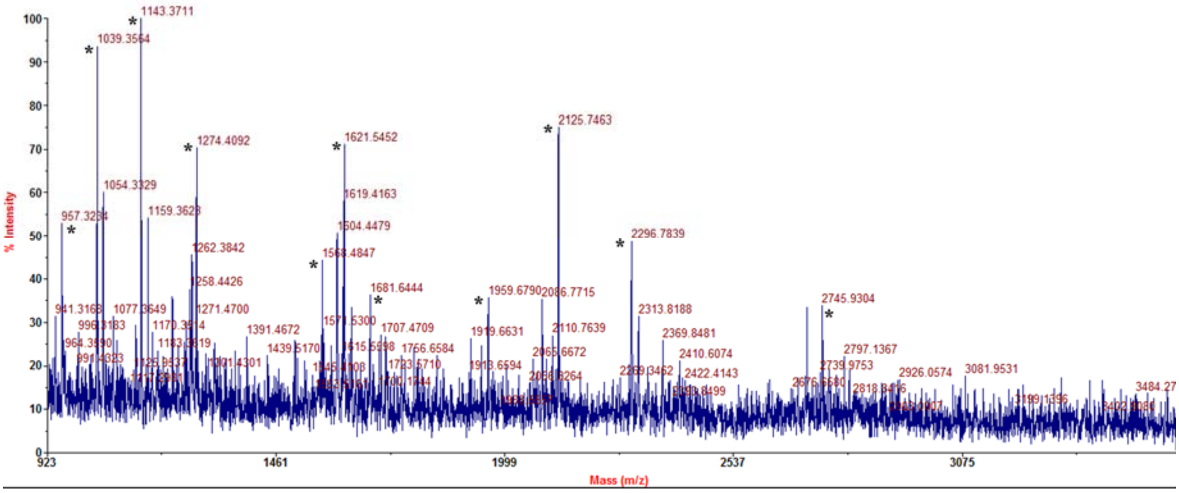

B. Peptides detected by MALDI-TOF-MS

Peptide	Amino acid sequence	$[M+H]^+$	Matched
125 - 139	IPFNSHEGGCGAAMR	1603.4630	+
47 - 165	FPHPSQLDTLIQVSIESGR	2123.7660	+
166 - 191	MTHHHPTGYLGSLASALFTAYAVNGK	2744.9210	+
209 - 219	AYVTQSGYFVK	1262.3950	+
246 - 257	SAPVFPKPFGVK	1273.4220	+
327 - 339	GVNPSNYEKLEYR	1568.4850	+

C. Matched peptides shown in **Bold Red**

1 MGGGLIERYV AAMVLSAAGD TLGYFNGKWE FLRDGEKIHR QLAQMGDLEA
51 IDVAQWRVSD DTIMHLATAE ALMEAGSSPD LPQLYSLLAK HYRDCMGDMD
101 GRAPGGACMQ NAMQLDPDRA DGWR**IPFNSH EGGCGAAMR**A MCIGLR**FPHP**
151 **SQLDTLIQVS IESGRMTHHH PTGYLGSLAS ALFTAYAVNG K**SPRQWGKGL
201 MEVLPEAK**AY VTQSGYFVK**E NLQHWSYFEK EWEKYLELRG ILDGK**SAPVF**
251 **PKPFGVK**ERD QFYIEVSYSG WGGSSGHDAP MIAYDALLAA GDSWKELAHR
301 AFFHGGDSDS TATIAGCWWG VMHGFK**GVNP SNYEKLEYR**Q RLEEAGRALY
351 SLGSKEDTIL GP

A. MALDI-TOF MS spectrum (spot 15)

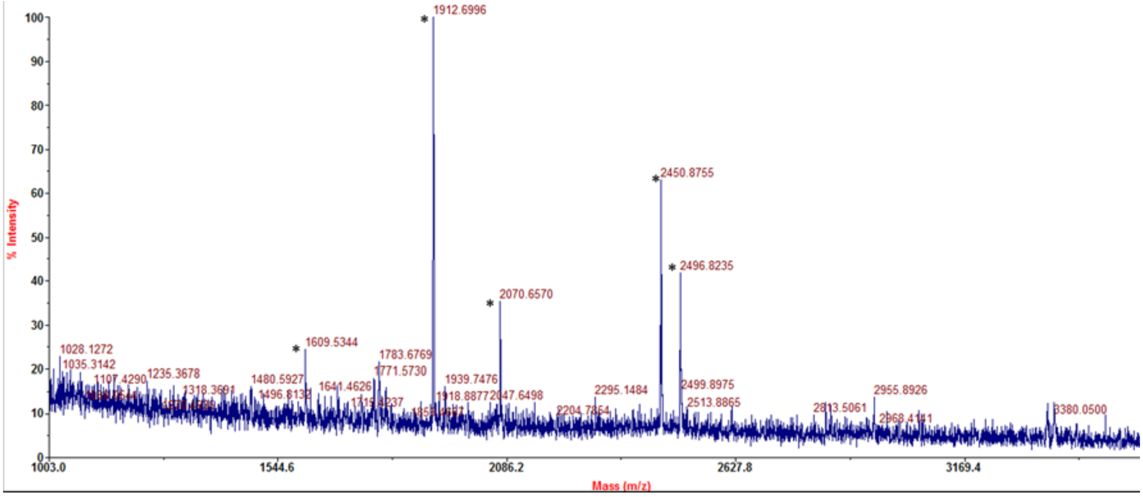

B. Peptides detected by MALDI-TOF-MS

Peptide	Amino acid sequence	$[M+H]^+$	Matched
117 - 138	HVSSGSFPPSTNEHVKEDLNLR	2449.8590	+
174 – 190	HNIQFSSFDIFSDEEVR	2069.6540	+
174 - 194	HNIQFSSFDIFSDEEVRQGLK	2495.8360	+
268 - 292	QILEILNSTGVEYETFDILEDEEVR	2953.9430	+
268 - 296	QILEILNSTGVEYETFDILEDEEVRQGLK	3380.0590	+
297 - 310	TFSNWPTYPQLYVR	1771.6320	+

C. Matched peptides shown in Bold Red

1 MAAGAAEAAE AAVAVVEVGS ARQFEELLRL KTKSLLVVHF WAPWAPQCVQ
51 MNDVMAELAK EHPHVSFVKL EAEAVPEVSE KYEISSVPTF LFFKNSQKVD
101 RLDGAHAPEL TKKVQR**HVSS GSFPPSTNEH VKEDLNLR**LK KLTHAAPCML
151 FMKGTPQEPR CGFSKQMVEI LHK**HNIQFSS FDIFSDEEVR QGLK**TYSNWP
201 TYPQLYVSGE LIGGLDIIKE LEASEELDTI CPKAPKLEER LKVLTNKASV
251 MLFMKGNKQE AKCGFSK**QIL EILNSTGVEY ETFDILEDEE VRQGLKTFSN**
301 **WPTYPQLYVR** GDLVGGLDIV KELKDNGELL PILKGEN

A. MALDI-TOF MS spectrum (spot 17)

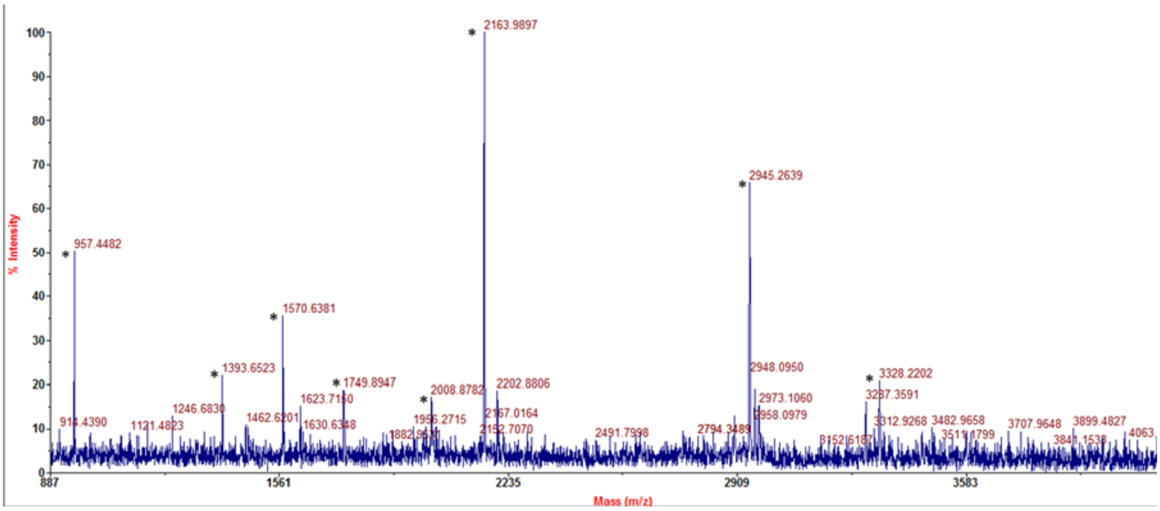

B. Peptides detected by MALDI-TOF-MS

Peptide	Amino acid sequence	$[M+H]^+$	Matched
52 - 68	QVELALWDTAGQEDYDR	2008.8170	+
69 - 98	LRPLSYPDTDVILMCFSVDSPDSLENIPEK	3450.3750	+
105 - 119	HFCPNVPIILVANKK	1749.8950	+
165 - 176	EGVREVFETATR	1393.6500	+
183 - 194	YGSQNGCINCCK	1460.6130	+

C. Matched peptides shown in Bold Red

1 MAAIRKKLVV VGDGACGKTC LLIVFSKDEF PEVYVPTVFE NYVADIEVDG
51 K**QVELALWDT AGQEDYDRLR PLSYPDTDVI LMCFSVDSPD SLENIPEK**WV
101 PEVK**HFCPNV PIILVANKK**D LRSDEHVRTE LARMKQEPVR TDDGRAMAVR
151 IQAYDYLECS AKTK**EGVREV FETATR**AALQ KR**YGSQNGCI NCCK**VL

A. MALDI-TOF MS spectrum (spot 20)

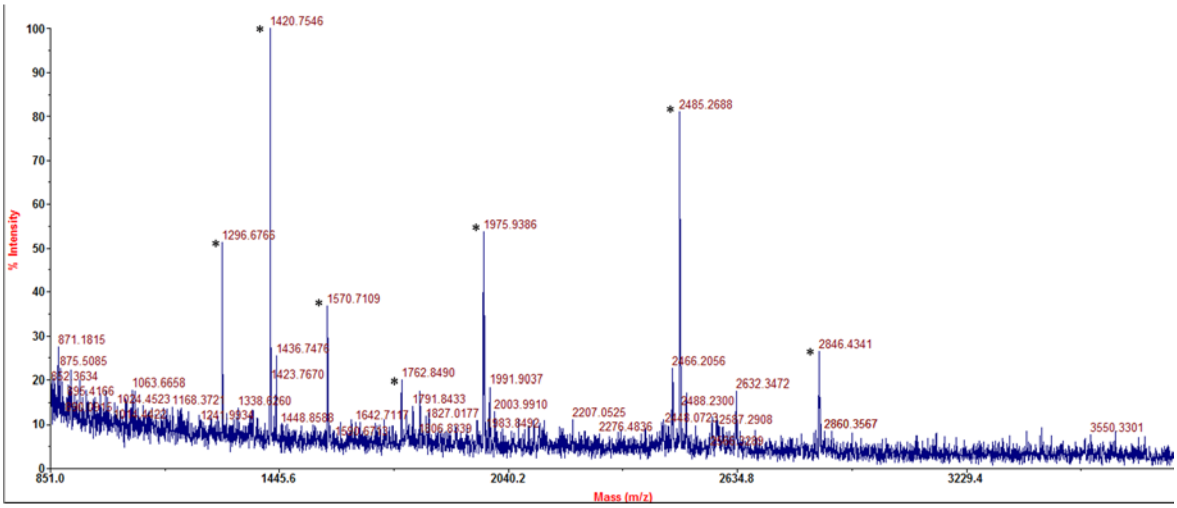

B. Peptides detected by MALDI-TOF-MS

Peptide	Amino acid sequence	$[M+H]^+$	Matched
22 - 44	AAPAAYDTSDTHLQILGKPVMER	2484.2640	+
22 - 44	AAPAAYDTSDTHLQILGKPVMER	2500.2800	+
45 - 60	WETPYMHSLAAAAASR	1761.8570	+
64 - 77	VLEVGFGMAIAASR	1420.7600	+
64 - 77	VLEVGFGMAIAASR	1436.7510	+
78 - 98	VQQAPIKEHWIIECNDGVFQR	2567.3070	+
180 - 204	SKYTDITAMFEETQVPALLEAGFQR	2845.4300	+
182 - 204	YTDITAMFEETQVPALLEAGFQR	2630.3210	+
205 - 221	ENICTEVMALVPPADCR	1974.9330	+
205 - 221	ENICTEVMALVPPADCR	1990.9130	+
222 - 236	YYAFPQMITPLVTKH	1808.9350	+

C. Matched peptides shown in Bold Red

1 MSSSAASPLF APGEDCGPAW R**AAPAAYDTS DTHLQILGKP VMERWETPYM**
51 **HSLAAAAASR** GGR**VLEVGFG MAIAASRVQQ APIKEHWIIE CNDGVFQR**LQ
101 NWALKQPHKV VPLKGLWEEE APTLPDGHFD GILYDTYPLS EETWHTHQFN
151 FIKTHAFRLL KPGGILTYCN LTSWGELMK**S KYTDITAMFE ETQVPALLEA**
201 **GFQRENICTE VMALVPPADC RYYAFPQMIT PLVTKH**

A. MALDI-TOF MS spectrum (spot 21)

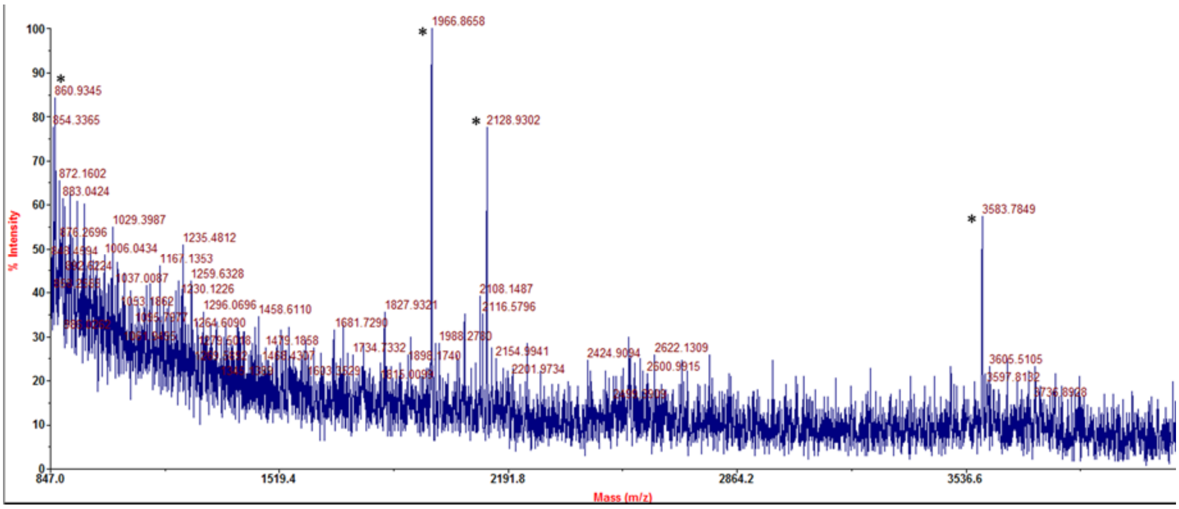

B. Peptides detected by MALDI-TOF-MS

Peptide	Amino acid sequence	$[M+H]^+$	Matched
43 - 73	VPFALTSDSNMPEDYPDQFDDVMDFIQATIK	3581.7640	+
43 - 74	VPFALTSDSNMPEDYPDQFDDVMDFIQATIKR	3737.8640	+
115 - 137	NRGCVLTAIHLNVTDLGLGYETK	2544.3390	+

C. Matched peptides shown in Bold Red

1 MKLWDVVAVC LVLLHTASAF PLPAGKRLLE APAEDHSLGH RR**VPFALTSD**
51 **SNMPEDYPDQ FDDVMDFIQA TIKR**LKRSPD KQAAALPRRE RNRQAAAASP
101 ENSRGKGRRG QRGK**NRGCVL TAIHLNVTDL GLGYETK**EEL IFRYCSGSCE
151 AAETMYDKIL KNLSRSRRLT SDKVGQACCR PVAFDDDLSF LDDSLVYHIL
201 RKHSAKRCGC I

A. MALDI-TOF MS spectrum (spot 22)

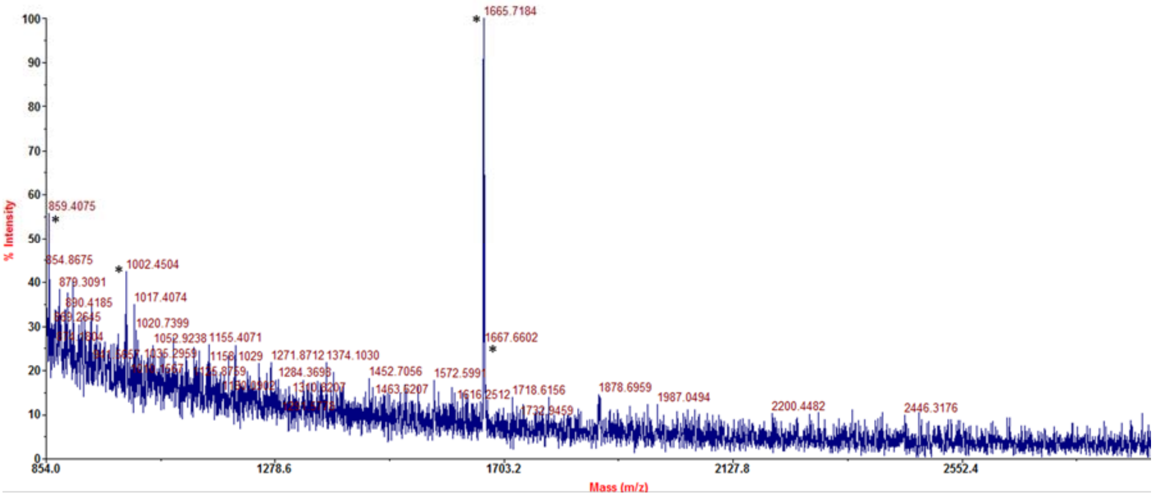

B. Peptides detected by MALDI-TOF-MS

Peptide	Amino acid sequence	$[M+H]^+$	Matched
12 - 25	SISTIINVFHQYSR	1664.6990	+
45 - 52	DLPNFLKR	1002.4170	+
87 - 94	LIFACHEK	1017.3830	+
87 - 101	LIFACHEKLHENNPR	1877.7230	+
95 - 101	LHENNPR	879.2710	+

C. Matched peptides shown in Bold Red

1 MAAKTGSQLE R**SISTIINVF HQYSR**KYGHP DTLNKAEFKE MVNK**DLPNFL**
51 **KR**EKRNENLL RDIMEDLDTN QDNQLSFEEC MMLMGK**LIFA CHEKLHENNP**
101 **R**GHDHSHGKG CGK

A. MALDI-TOF MS spectrum (spot 23)

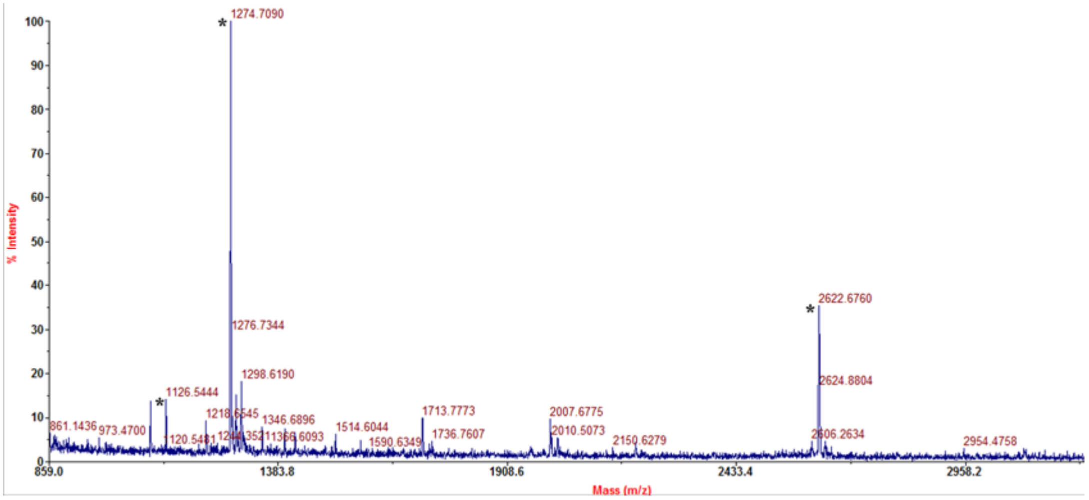

B. Peptides detected by MALDI-TOF-MS

Peptide	Amino acid sequence	$[M+H]^+$	Matched
19 - 31	VNPDDVGGEALGR	1298.6220	+
32 - 41	LLVVYPWTQR	1274.7280	+
42 - 60	YFDSFGDLSSASAIMGNPK	2006.6910	+
42 - 60	YFDSFGDLSSASAIMGNPK	2022.6260	+
67 - 77	KVINAFNDGLK	1218.6790	+
68 - 77	VINAFNDGLK	1090.6130	+
84 - 96	GTFAHLSELHCDK	1514.6160	+
84 - 105	GTFAHLSELHCDKLHVDPENFR	2621.8500	+
97 - 105	LHVDPENFR	1126.5620	+
106 - 121	LLGNMIVIVLGHHLGK	1713.9140	+
106 - 121	LLGNMIVIVLGHHLGK	1729.8850	+
106 - 133	LLGNMIVIVLGHHLGKEFTPCAQAAFQK	3092.3530	+
122 - 133	EFTPCAQAAFQK	1397.6320	+
134 - 147	VVAGVASALAHKYH	1422.7250	

C. Matched peptides shown in Bold Red

1 MVHLTDAEKA AVNGLWGK**VN PDDVGGEALG RLLVVYPWTQ RYFDSFGDLS**
51 **SASAIMGNPK** VKAHGK**KVIN AFNDGLK**HLD NLK**GTFAHLS ELHCDKLHVD**
101 **PENFRLLGNM IVIVLGHHLG KEFTPCAQAA FQKVVAGVAS ALAHKYH**

A. MALDI-TOF MS spectrum (spot 25)

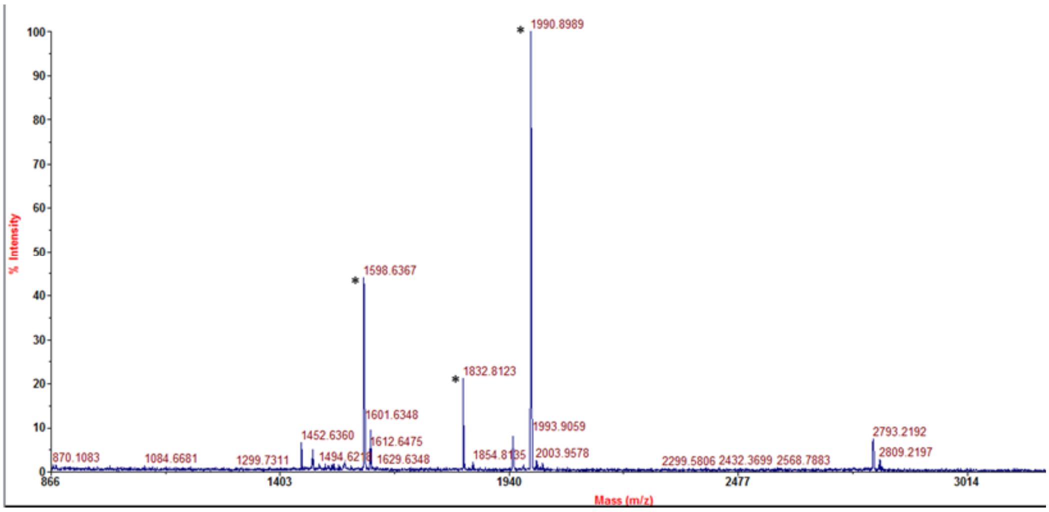

B. Peptides detected by MALDI-TOF-MS

Peptide	Amino acid sequence	$[M+H]^+$	Matched
2 - 19	VNPTVFFDITADGEPLGR	1947.8990	+
20 - 31	VCFELFADKVPK	1452.6270	+
56 - 69	IIPGFMCQGGDFTR	1598.6380	+
77 - 91	SIYGEKFEDENFILK	1831.8070	+
92 - 118	HTGPGILSMANAGPNTNGSQFFICTAK	2807.2400	+
119 - 131	TEWLDGKHVVFGK	1515.6910	+
132 - 144	VKEGMSIVEAMER	1478.6100	+
132 - 144	VKEGMSIVEAMER	1494.6200	+
134 - 144	EGMSIVEAMER	1251.4420	+
155 - 164	KITISDCGQL	1134.4300	+

C. Matched peptides shown in Bold Red

1 M**VNPTVFFDI TADGEPLGRV CFELFADKVP K**TAENFRALS TGEKGFGYKG
51 SSFHR**IIPGF MCQGGDFTR**H NGTGGK**SIYG EKFEDENFIL KHTGPGILSM**
101 **ANAGPNTNGS QFFICTAKTE WLDGKHVVFG KVKEGMSIVE AMER**FGSRNG
151 KTSK**KITISD CGQL**
